# Molecular Modeling of the Human Hemoglobin-Haptoglobin Complex Sheds Light on the Protective Mechanisms of Haptoglobin

**DOI:** 10.1371/journal.pone.0062996

**Published:** 2013-04-26

**Authors:** Chanin Nantasenamat, Virapong Prachayasittikul, Leif Bulow

**Affiliations:** 1 Center of Data Mining and Biomedical Informatics, Faculty of Medical Technology, Mahidol University, Bangkok, Thailand; 2 Department of Clinical Microbiology and Applied Technology, Faculty of Medical Technology, Mahidol University, Bangkok, Thailand; 3 Pure and Applied Biochemistry, Department of Chemistry, Lund University, Lund, Sweden; King's College, London, United Kingdom

## Abstract

Hemoglobin (Hb) plays a critical role in human physiological function by transporting O_2_. Hb is safe and inert within the confinement of the red blood cell but becomes reactive and toxic upon hemolysis. Haptoglobin (Hp) is an acute-phase serum protein that scavenges Hb and the resulting Hb-Hp complex is subjected to CD163-mediated endocytosis by macrophages. The interaction between Hb and Hp is extraordinarily strong and largely irreversible. As the structural details of the human Hb-Hp complex are not yet available, this study reports for the first time on insights of the binding modalities and molecular details of the human Hb-Hp interaction by means of protein-protein docking. Furthermore, residues that are pertinent for complex formation were identified by computational alanine scanning mutagenesis. Results revealed that the surface of the binding interface of Hb-Hp is not flat and protrudes into each binding partner. It was also observed that the secondary structures at the Hb-Hp interface are oriented as coils and α-helices. When dissecting the interface in more detail, it is obvious that several tyrosine residues of Hb, particularly β145Tyr, α42Tyr and α140Tyr, are buried in the complex and protected from further oxidative reactions. Such finding opens up new avenues for the design of Hp mimics which may be used as alternative clinical Hb scavengers.

## Introduction

Hemoglobin (Hb) is a ubiquitous protein found in all kingdoms of life, i.e. in archaea, bacteria, fungi, protists, plants and animals [1]. It can inherently bind gaseous diatomic ligands such as O_2_, CO and NO via its heme prosthetic group, which is bound to the protein via the axial histidine ligands. Human HbA is a tetrameric structure comprising of two αβ dimers. The globin-fold of each monomeric chain is comprised of eight helices with A, E and F helices stacking on top of B, G and H helices. Such structural topology is known as the 3-on-3 α-helical sandwich-fold while the 2-on-2 α-helical sandwich-fold, which is found in truncated Hb, places the B and E helices on top of the G and H helices. This globin-fold harbours the heme prosthetic group via coordination to the axially ligated histidine residues at the proximal and distal locations on the F and E helices, respectively.

Inside the erythrocyte, Hb is in a tetrameric form where it is present in the reduced and nontoxic state owing to a wide range of protective enzymatic systems [2]–[4]. However, upon intravascular hemolysis, caused e.g. by hemolytic anemias, autoimmune transfusion reactions, preeclampsia or intraventricular hemohorrhage and subarachnoidal bleeding [3], it separates easily into agile dimeric forms that quickly can cause oxidative toxicity. Haptoglobin (Hp), which was first identified by Polonovski and Jayle in 1938 [5], is an Hb scavenger that prevents many of the toxic effects caused by Hb. Hp is just like Hb, a tetrameric protein of four chains with molecular weights of 9 and 33 kDa, respectively, for each of the two α-chains and β-chains. This form of haptoglobin is called Hp 1-1, but the protein is also present in several different oligomeric structures which harbour a different α-chain [3]. Hp is an acute phase-plasma glycoprotein of the α_2_-globulin fraction found in plasma of humans and mammals [6]. Owing to its innate Hb binding property, Hp can effectively protect renal functions as well as provide other valuable physiological properties such as acting as an anti-inflammatory agent, antioxidant, angiogenic promoter, immune cell regulator and therapeutic agent of sickle cell disease [7]. This can be explained on the molecular level, since Hp counters the deleterious effects of toxic cell-free Hb by forming a very strong and specific Hb-Hp complex. This is today considered to be one of the strongest molecular interactions (*K*_d_  = 10^−14^ M) known in nature [8]. The Hb-Hp conjugate is then rapidly bound to the macrophage membrane protein CD163 followed by its engulfment and degradation. The utilization of this specific Hp-Hb interaction with CD163 represents an important potential clinical tool for modulating the physiological status of macrophages, e.g. by controlling an inflammatory response.

The endeavors to get a better understanding on how Hb and Hp bind have resulted in a series of experimental investigations, which have slowly accumulated over the years. Such efforts have shed light on the Hb and Hp-binding sites on each protein. The Hp-binding site encompasses residues 121–135 [9] and 139–141 [10] of Hb α-chain and residues 11–25, 37 and 131–146 [11] of Hb β-chain. Likewise, the Hb-binding site comprises of residues 9-10, 128–131, 136–137 [12], 136, 218 [13], 137 [14] and 234–264 [15] of the Hp β-chain. Surprisingly, residues 1–70 on Hp α-chain [15] have also been suggested.

Preliminary information on the structural insights of Hb-Hp complex have also previously been provided by electron microscopic studies, which indicated that the Hb dimer interacts with Hp β-chain forming an angle of 127° relative to the axis of Hp [16]. In spite of this, the structural detail of this complex is not fully understood. Almost two decades later, Przybylska et al. [17] attempted to crystallize the Hb-Hp complex, however the crystals did not diffract well enough for data collection, which largely shortcut further investigations. Molecular modeling has frequently been instrumental for dissecting a range of biological phenomena [18]–[23] and is ever more important in contributing to better understanding of protein-protein interactions [24]–[27]. In this study, we have therefore examined the structural basis and binding modalities governing the interaction between Hb and Hp 1–1. This was achieved by first obtaining an ensemble of energetically stable conformers of Hb and Hp from the last 10 ns of molecular dynamics simulations. Such structures were subsequently docked using an extensive set of biochemical, structural and biophysical data as restraints to drive the docking calculations. Hot-spot residues of each binding partner were identified by means of computational alanine scanning mutagenesis. We were also specifically studying the structural details of the Hb tyrosine residues in the complex since these are known to be involved in toxic redox reactions [28].

## Materials and Methods

### Preparation of protein structures

The crystal structure of human Hb containing a single αβ dimer was obtained from the Protein Data Bank (accession number 2DN1) [29] while the homology models of haptoglobin variant 1 was obtained from the Protein Model DataBase (accession number PM0075389) [30]. The N-terminal valine residues of α and β subunits of hemoglobin, which were not present in the structure, were modeled in using PyMol [31]. Coordinates of the heteroatoms, toluene and oxygen, were removed from the structure. The protonation state of histidines and other ionizable residues were assigned to the hemoglobin structure according to the structure of human hemoglobin determined at pH 6.7 via neutron crystallography [32], [33]. For comparative purposes, the protonation states of ionizable residues in Hp were determined at pH 6.7 by the PDB2PQR web server [34] according to the PROPKA method [35]. Hydrogen atoms were then added to the structures using the pdb2gmx utility as provided by the GROMACS simulation package.

### Molecular dynamics simulation

Molecular dynamics (MD) simulations were performed using the GROMOS 43A1 force field [36] with GROMACS version 4.0.5 [37]. A preliminary energy minimization of the protein structures was performed *in vacuo* in order to remove unfavorable van der Waals contacts. The minimization was performed for a maximum of 5,000 steps using the steepest descent algorithm. The convergence criterion of the energy minimization is reached when the maximum force is less than 10 kJ mol^−1^ nm^−1^ or until no significant improvement can be attained between-steps.

The protein was placed at the center of a rhombic dodecahedral box solvated with single point charge (SPC) water molecules. The minimum distance between the protein and the wall of the simulation box was set to 1.5 nm. Periodic boundary conditions were applied in order to solve the issue of surface effects as well as to simulate bulk systems. Physiological ionic strength of 0.15 M NaCl was applied as counter-ions in order to equilibrate the system. Another round of energy minimization was performed on the solvated system using the procedures described above.

Two phases of equilibration were conducted under constant Number of particles, Volume and Temperature (NVT) followed by keeping the Number of particles, Pressure and Temperature (NPT) constant. The NVT ensemble was performed in two stages where in the first stage, the system was slowly heated over a time of 200 ps to bring the temperature to 200 K by randomly assigning initial velocities taken from Maxwell−Boltzmann distribution. Positional restraints were applied on the protein as to allow solvent molecules to relax around the structure. In the second stage, positional restraints were lifted and the system was coupled to a heat bath at 300 K using the Berendsen [38] thermostat and allowed to equilibrate for 200 ps. In an NPT ensemble, the Berendsen barostat was used for controlling the pressure to 1 atm with a compressibility of 4.5×10^−5^ bar^−1^. Time constants for controlling the temperature (τ_T_) and pressure (τ_P_) were set to 0.1 and 1 ps, respectively.

The production run was performed for 20 ns at a temperature and pressure of 300 K and 1 atm, respectively, according to the aforementioned settings and simulation. Snapshots were collected every 1 ns using an integration time step of 2 fs. For all MD simulations, the reaction field method [39] was used to treat long-range electrostatic interactions applying cutoff distances of 1.4 nm for both Coulomb and van der Waals interactions and a dielectric constant of 78 was applied for electrostatic interactions beyond 1.4 nm. The short-range neighbor list of 0.9 nm was used. Bond lengths were constrained using the Linear Constraint Solver (LINCS) algorithm [40].

Analysis of MD trajectories was performed using utilities within the GROMACS package.

### Protein-protein docking calculation

The interaction of Hb and Hp was simulated by means of protein-protein docking calculation using HADDOCK server [41], [42], which is an approach that incorporates biochemical and biophysical data to drive the docking process. HADDOCK achieves this by employing ambiguous interaction restraints that are known about the binding interface. Thus, residues that have previously been determined to be involved at the Hb-Hp binding interface were designated as active residues if they are also solvent exposed. This surface exposure was determined by GETAREA using criteria of greater than 40% solvent accessibility. Residues within a vicinity of 6.5 Å from active residues were designated as passive residues if they are also solvent exposed. Such lists of active and passive residues (as shown in Table 1) were used as ambiguous interaction restraints. Unambiguous restraints between the iron atom and nitrogen atoms of coordinating histidine residues were also defined. Finally, an ensemble of structures from the last 10 ns of MD simulation was cross-docked to give rise to a matrix of 10×10 different combinations. A positional RMSD cutoff of 7.5 Å was used for clustering docking solutions; it should be noted that only clusters with at least 5 structures were considered.

**Table 1:** Summary of interaction restraints used in protein-protein docking.

	Residues
*Ambiguous interaction restraints*	
Hb	
Active residues	αLys127, αAla130, αThr134, αLys139, αArg141, βThr12, βAla13, βGly16, βLys17, βAsn19, βGlu22, βTrp37, βAla135, βAsn139, βHis143, βLys144, βTyr145, βHis146
Passive residues	αVal1, αSer3, αHis89, αArg92, αSer138, βLys8, βSer9, βPro36, βArg40, βSer72, βThr87, βGlu90, βLys95, βLeu96, βHis97, βGlu101, βArg104
Hp	
Active residues	βLys9, βPhe129, βLys130, βPhe131, βHis134, βAsp144, βAsp146, βArg150, βThr156, βVal157, βPro158, βGlu159, βLys160, βLys161, βThr162, βLeu203, βGlu204, βGlu205, βAsp206, βLys218, βVal222
Passive residues	αAsn36, αLys76, αAsn77, βLeu6, βAla8, βAsn23, βThr61, βGlu99, βLys109, βVal114, βAsn126, βAla127, βHis151, βPro163, βSer183
Hp_β_ *	
Active residues	βLys9, βLys130, βPhe131, βAsp144, βAsp146, βArg150, βThr156, βVal157, βGlu159, βLys160, βLys161, βThr162, βLeu203, βGlu204, βGlu205, βAsp206, βVal222, βAla223
Passive residues	βHis5, βLeu6, βAla8, βVal64, βGlu99, βVal114, βPro163, βLys164, βLeu173, βSer183, βSer184, βGln186, βGlu187
*Unambiguous interaction restraints*	
Hb	^Heme^Fe-N_ε_^αHis87^
	^Heme^Fe-N_ε_^βHis92^

* Haptoglobin without α chain.

### Analysis of protein-protein interface

PISA [43] was used to analyze the Hb-Hp structures and binding interfaces from the top ten Hb-Hp docking models. The size of the protein-protein binding interface was calculated from the solvent accessible surface area (SASA) according to the following equation:
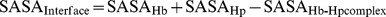



PROTORP [44] was then employed to assess the geometrical parameters of the protein complex. Gap volume index is a parameter that accounts for shape complementarity of the interface and is calculated according to the following equation:
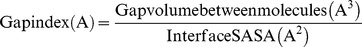



### Hot spot calculation

Computational alanine scanning mutagenesis was performed to identify hot spots of protein-protein interaction using the graphical interface plugin [45] of FoldX [46] empirical force field for Yasara [47]. FoldX is an empirical force field that was formulated by analyzing 1,000 point mutations from 82 protein-protein complexes. The FoldX energy function accounts for several thermodynamic terms that are known to be important for protein stability, which includes van der Waals interactions, solvation effects, hydrogen bonds, water bridges, electrostatic and entropy effects for the backbone and the side-chain.

In this study, Hb-Hp interfacial residues were sequentially mutated to alanine and their energetic difference were calculated as shown in the following equation:
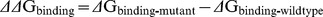
where 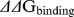 represents the binding energy difference of mutant and wild-type, 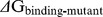 represents the binding energy of mutant and 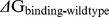 represents the binding energy of wild-type.

The binding energy of Hb and Hp is calculated as the difference of the complex and its respective monomers as follows:
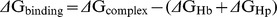
where 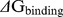 is the binding energy for either the mutant or wild-type, 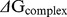 is the energy of Hb-Hp complex, 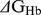 is the energy of Hb and 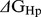 is the energy of Hp. Residues giving rise to 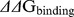 ≥1.5 kcal/mole were identified as potential hot spots.

## Results and Discussion

Both human Hb and Hp 1-1 are tetramers comprised of two αβ dimers where the former adopts a 3-on-3 α-helical globin fold and the latter takes the form of a linear dumbbell-shaped conformation [16]. The two αβ-subunits of Hp form the aforementioned linear conformation as a result of the disulfide bond cross-linkage of the two α-chains of the αβ-subunits. An electron microscopic study by Wejman et al. [16] provided early insights on the molecular topology of the Hb-Hp complex where one tetrameric α_2_β_2_ molecule of Hp can interact with two αβ dimer of the tetrameric α_2_β_2_ Hb, particularly, the β-chain of Hp can interact with the αβ dimer of Hb. This study aims to further elucidate the interaction of Hb-Hp complex at the molecular level through the use of molecular dynamics and protein-protein docking methods, particularly focusing on how Hp can protect against toxic Hb radical formation.

### Preparation of Hb and Hp structures

This study employs the crystal structure of a single αβ dimer of Hb and the homology modeled structure of the αβ dimer of Hp 1-1. The protein structures were then prepared for subsequent MD and docking simulations by removing heteroatoms as well as assigning appropriate protonation states for histidines and ionizable residues.

To discern the influence of the Hp α-chain on Hb-Hp interaction, a structural model of Hp was prepared in the absence of the α-chain and the resulting structure will be called Hp β-chain and abbreviated as Hp_β_ from hereon. This structure was subjected to the same computational protocol as the other proteins.

### Molecular dynamics of Hb and Hp/Hp_β_

**Figure 1:**
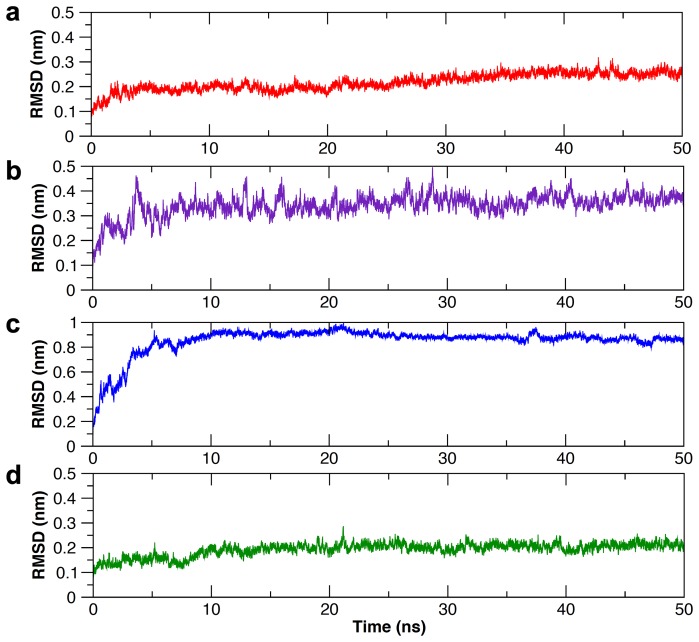
Structural fluctuation from MD simulation as described in terms of RMSD as a function of time for Hb (a), Hp (b), Hp_α_ (c) and Hp_β_ (d).

**Figure 2:**
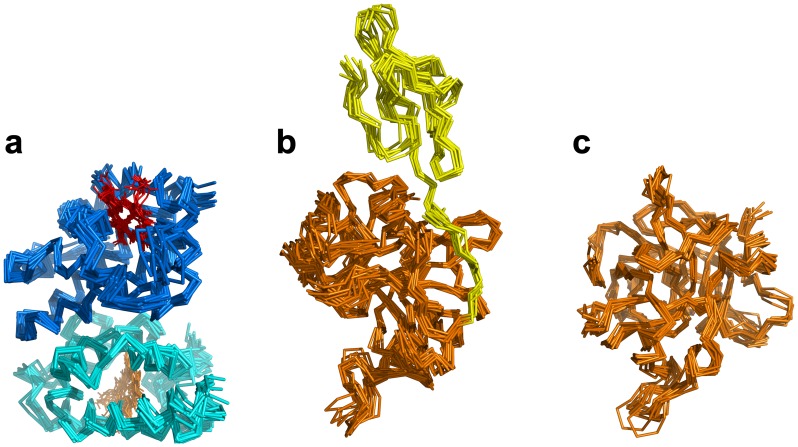
Ensemble of structures from the last 10 ns of MD simulation for Hb (a), Hp (b) and Hp_β_ (c).

Molecular dynamics was employed to allow conformational relaxation of the structures before subjecting them to further protein-protein docking calculations. From Figure 1, it can be seen that all structures displayed an initial structural rearrangement that is followed by convergence to an RMSD plateau indicating minimal structural fluctuations during the latter part of the simulation. The overall structural fluctuation of Hb (Figure 1a) was observed to be less than that of Hp (Figure 1b) with an RMSD of 0.2 nm and 0.35 nm, respectively, which is to be expected as the protein structure of the former was obtained from X-ray crystallographic experiment (with a high resolution of 1.25 Å) while the latter was obtained from homology modeling. Furthermore, upon splitting the Hp structure into two separate domains, namely α- and β-chains (Figures 1c and 1d), it was observed that both chains displayed significantly different structural fluctuations with an RMSD of 0.9 and 0.2 nm, respectively, suggesting the former to be significantly more flexible than the latter chain. It should be noted that the large movement observed for the C-terminus of the α-chain and its apparently greater flexibility is perhaps not completely unexpected, due to the fact that the α-chain is truncated (13 residue missing) and to the lack of the disulfide bond between Cys15 of the α-chain and Cys15 of the partner α-chain that is missing in the model. It is also worthy to note that the two α-chains are in tight contact to each other. An ensemble of structures from the last 10 ns of MD simulation for Hb, Hp and Hp_β_ were then used for further docking calculations (Figure 2).

### Docking Hb to Hp/Hp_β_

**Figure 3:**
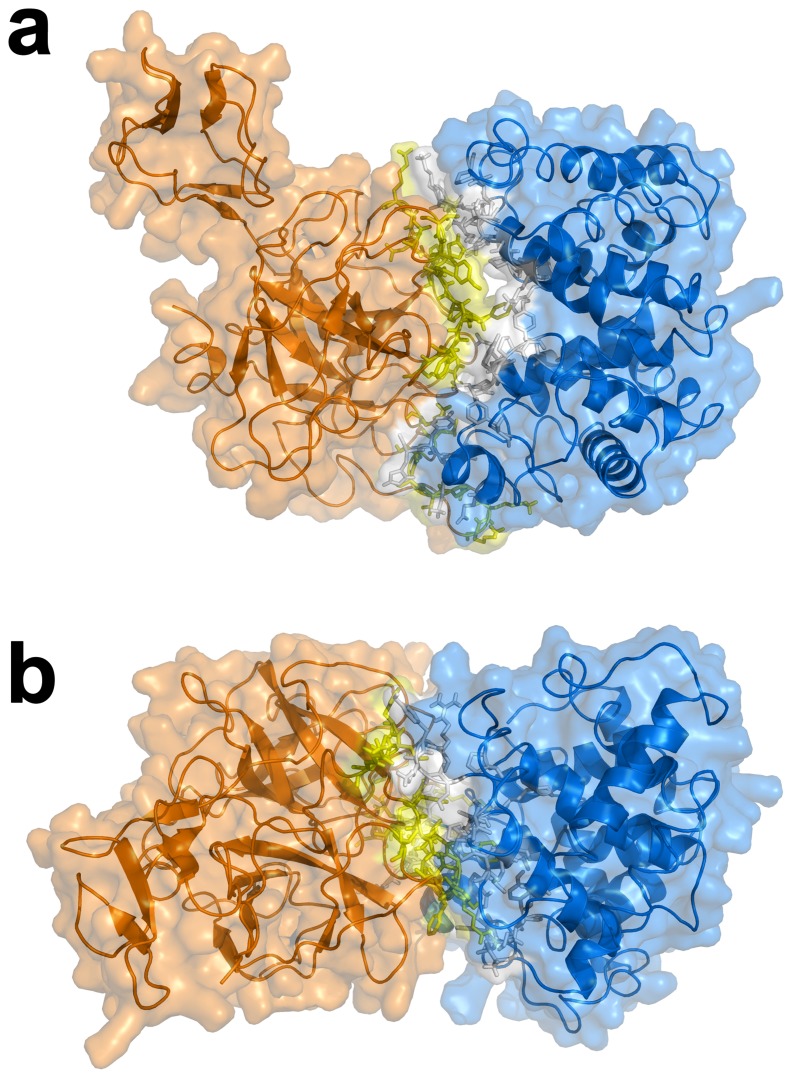
Top ranked structure of Hb-Hp from the best cluster of docking simulation is shown from the side (a) and top (b) view.

**Figure 4:**
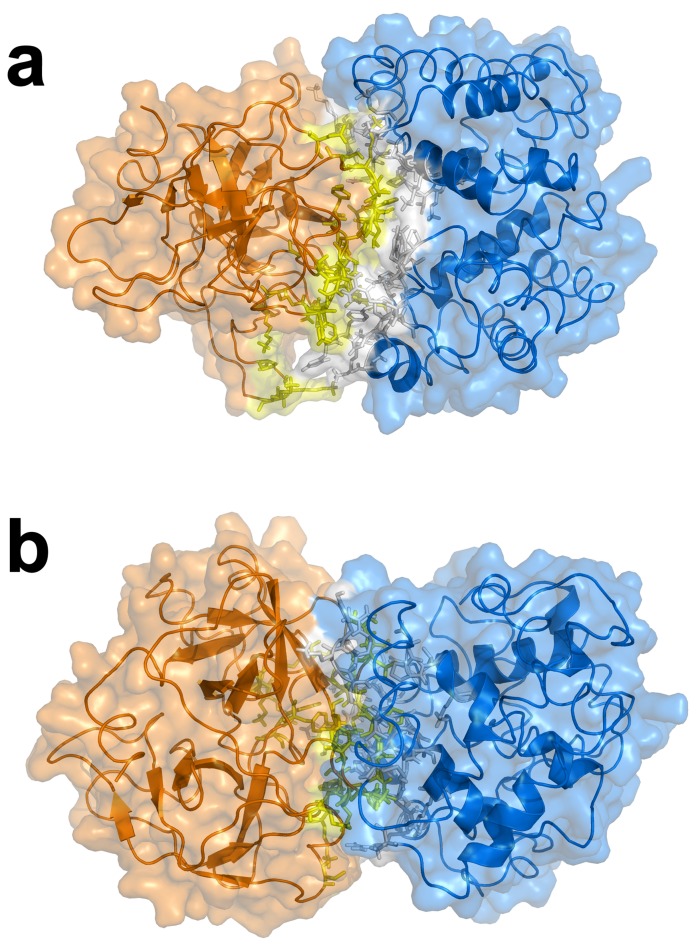
Top ranked structure of Hb-Hp_β_ from the best cluster of docking simulation is shown from the side (a) and top (b) view.

**Figure 5:**
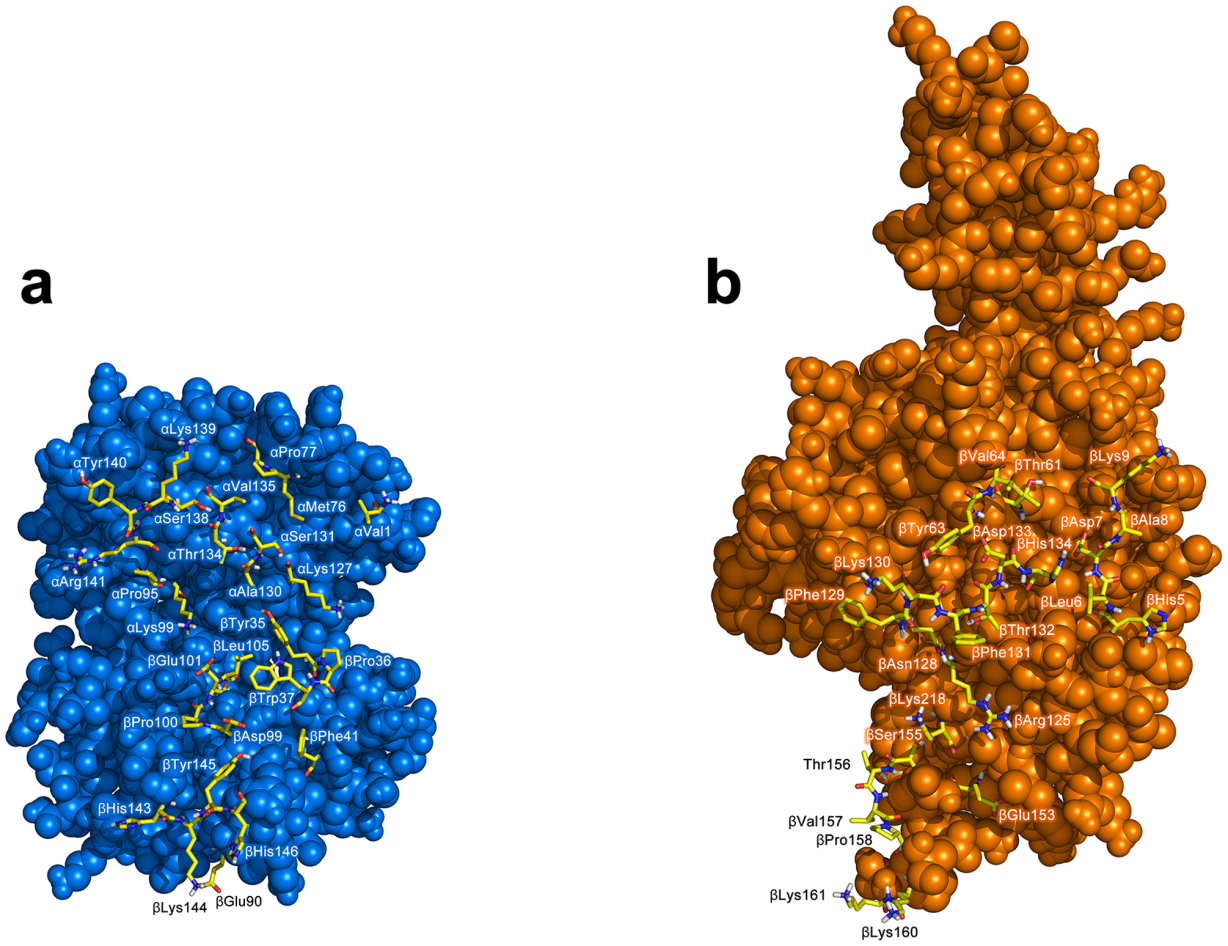
Residues at the binding interfaces of Hb and Hp from docking complexes of Hb-Hp are displayed as yellow colored sticks.

**Figure 6:**
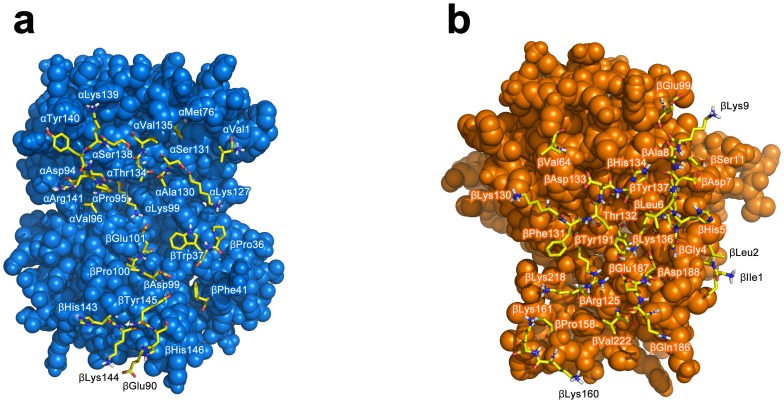
Residues at the binding interfaces of Hb and Hp_β_ from docking complexes of Hb-Hp_β_ are displayed as yellow colored sticks.

The biochemical, structural and biophysical data obtained from previous investigations that had assimilated over the years were used as ambiguous interaction restraints to drive the docking protocol using HADDOCK. This is comprised of 18, 21 and 18 active residues for Hb, Hp and Hp_β_, respectively, while there are 17, 15 and 13 passive residues for Hb, Hp and Hp_β_, respectively. A cut-off value of 7.5 Å was used for clustering solutions of the protein-protein complexes, which gave rise to a total of 281 and 259 structures in 17 and 16 clusters, respectively, for Hb-Hp and Hb-Hp_β_. It should be noted that the largest cluster (and also the top ranking cluster) had 87 and 89 structures, respectively, for Hb-Hp and Hb-Hp_β_ with a positional RMSD of 2.35 and 2.53 Å amongst the members, thus indicating moderately low structural variability within the clusters. Selection of the best representative cluster was made on the basis of the lowest HADDOCK score, which was 13.43 and 31.76 for Hb-Hp and Hb-Hp_β_, respectively. It should be noted that the HADDOCK score represents a combination of energy terms that was calculated in relation to the buried surface area value. The top ranked structure for Hb-Hp and Hb-Hp_β_ from the best cluster is shown in Figures 3 and 4, respectively, from the side and top views. Residues at the binding interfaces of Hb-Hp and Hb-Hp_β_ are shown in Figures 5 and 6, respectively.

### Analysis of Hb-Hp and Hb-Hp_β_ binding interface

Putative interface residues identified by PISA in the top ten models of Hb-Hp indicated an average of 33.8±4.5 (ranging from 25 to 39) and 33.3±4.9 (ranging from 26 to 40) residues of Hb and Hp, respectively, at the binding interface. It was observed that the interface area occupied 1146.4±181 Å^2^ (ranging from 835.1 to 1389.9) or roughly 6.1 and 6.9% of the SASA of Hb and Hp with SASA of 16708.6±141.9 and 18848±145.4 Å^2^, respectively. Of all the putative interface residues identified by PISA, consensus residues found in seven of the top ten Hb-Hp models from the HADDOCK calculation are shown in Tables 2 and 3 for Hb-Hp and Hp_β_-Hp, respectively. The number of continuous interface residue segment for Hb-Hp was found to be 14±0.9 (ranging from 12 to 15) as calculated from PROTORP. The gap volume index of Hb-Hp was 5.1±1 Å, which is typical of non-obligate interaction (where each binding partner can exist individually as functional proteins whereas obligates cannot). Nooren and Thornton [42] noted that interfaces of transient complexes have small, planar and polar interface area as compared to those of homodimers. Planarity is a measure of the shape of the binding interface that is the flatness of an interface. The results show that the protein complex had a significantly less planar surface at the interface with a planarity value of 3.6±0.4 Å as compared to the average planarity of protein-protein interfaces of 3±0.5 Å [48]. This indicated that the protein interface is not flat and that there is protrusion into both binding partner. Results from PROTORP analysis pointed out that the secondary structures at the binding interface included coils and α-helices. Additionally, an estimate of the relative distribution of the polarity of surface exposed residue was carried out. Surface exposed residues were categorized into 3 classes: low polarity (Leu, Ile, Phe, Trp, Cys, Met, Val and Tyr), neutral polarity (Pro, Ala, Thr, Gly and Ser) and high polarity (His, Asp, Glu, Asn, Gln, Arg and Lys). We found that surface exposed residues of Hb and Hp monomers before complexation had 36, 67 and 123 residues belonging to the low, neutral and high polarity classes, respectively, or approximately 15.9%, 29.6% and 54.4%, respectively, of the residues. After Hb-Hp complex formation the buried residues or those right at the binding interface are composed of 5, 10 and 10 residues, respectively, corresponding to 20%, 40% and 40%, respectively, of the residues. This result corroborates the fact that an equal share of residues had neutral and high polarity at the binding interface.

**Table 2:** List of residues at the binding interface of Hb-Hp complex.*

Residue	Frequency	Residue	Frequency
*Hemoglobin α*		*Haptoglobin β*	
** Val1**	**8**	His5	9
** Met76**	**9**	Leu6	10
Pro77	7	Asp7	9
** Pro95**	**10**	Ala8	10
** Lys99**	**7**	Lys9	9
** Lys127**	**10**	Thr61	9
** Ala130**	**8**	Tyr63	9
** Ser131**	**9**	**Val64**	**10**
** Thr134**	**10**	**Arg125**	**9**
** Val135**	**10**	Asn128	9
** Ser138**	**10**	Phe129	10
** Lys139**	**10**	**Lys130**	**10**
** Tyr140**	**10**	**Phe131**	**10**
** Arg141**	**10**	**Thr132**	**9**
		**Asp133**	**9**
*Hemoglobin β*		**His134**	**10**
Tyr35	10	Glu153	8
** Pro36**	**9**	Ser155	8
** Trp37**	**10**	Thr156	10
** Phe41**	**7**	Val157	10
Glu90	9	**Pro158**	**10**
** Asp99**	**7**	**Lys160**	**10**
** Pro100**	**7**	**Lys161**	**8**
** Glu101**	**10**	**Lys218**	**10**
Leu105	9		
** His143**	**9**		
** Lys144**	**10**		
** Tyr145**	**10**		
** His146**	**10**		

* Residues in bold text are those also found in the Hb-Hp_β_ complex.

**Table 3:** List of residues at the binding interface of Hb-Hp_β_ complex. *

Residue	Frequency	Residue	Frequency
*Hemoglobin α*		*Haptoglobin β*	
** Val1**	**8**	Ile1	8
** Met76**	**8**	Leu2	10
Asp94	7	Gly4	7
** Pro95**	**10**	His5	10
Val96	7	Leu6	10
** Lys99**	**10**	Asp7	10
** Lys127**	**8**	Ala8	10
** Ala130**	**9**	Lys9	10
** Ser131**	**10**	Ser11	9
** Thr134**	**10**	**Val64**	**7**
** Val135**	**8**	Glu99	8
** Ser138**	**10**	**Arg125**	**10**
** Lys139**	**9**	**Lys130**	**10**
** Tyr140**	**8**	**Phe131**	**10**
** Arg141**	**10**	**Thr132**	**10**
		**Asp133**	**10**
*Hemoglobin β*		**His134**	**10**
** Pro36**	**8**	Lys136	10
** Trp37**	**10**	Tyr137	9
Arg40	10	**Pro158**	**8**
** Phe41**	**10**	**Lys160**	**10**
Asp94	8	**Lys161**	**7**
His97	10	Gln186	10
** Asp99**	**10**	Glu187	10
** Pro100**	**10**	Asp188	7
** Glu101**	**10**	Tyr191	10
Asn139	10	**Lys218**	**8**
Ala142	9	Val222	8
** His143**	**10**		
** Lys144**	**10**		
** Tyr145**	**10**		
** His146**	**10**		

* Residues in bold text are those also found in the Hb-Hp complex.

As for the Hb-Hp_β_ complex there was an average of 35.6±3.4 (ranging from 29 to 40) and 37.9±4.6 (ranging from 31 to 45) residues of Hb and Hp_β_, respectively. The interface area occupied 1353.3±195.2 Å^2^ (ranging from 1079.4 to 1668.8) or roughly 8.1 and 9.6% of the SASA of Hb and Hp with SASA of 16716.4±190.7 and 14157.2±224.5 Å^2^, respectively. The number of continuous interface residue segment for Hb-Hp_β_ was 13.2±1.9 (ranging from 10 to 15). The gap volume index of 3.8±0.6 Å for Hb-Hp_β_ is lower than that of Hb-Hp_,_ which is indicative of closer packing than that of the latter. The planarity of Hb-Hp_β_ is roughly similar to that of Hb-Hp with a value of 3.6±0.4 Å, also suggesting that the binding interface is not flat and may protrude into the respective binding partner. Similar to that of Hb-Hp_,_ the results also suggested that the secondary structures at the binding interface of Hb-Hp_β_ comprised of coils and α-helices. Furthermore, the relative distribution of the polarity of surface exposed residue revealed that before complexation there were 31, 67 and 92 residues belonging to the low, neutral and high polarity classes, respectively, or approximately 16.3%, 35.3% and 48.4%, respectively, of the total residues. After Hb-Hp_β_ complex formation the buried residues or those situated at the binding interface are composed of 6, 8 and 4 residues, respectively, corresponding to 27.3%, 36.4% and 18.2%, respectively, of the residues. This result indicated that the absence of the α-chain led to structural perturbation of the β-chain that influenced the binding interface by shifting the polarity of interface residues towards lower polarity.

### Identification of hot spot residues

Alanine scanning mutagenesis of protein-protein interfaces had indicated that the binding energy is not evenly distributed throughout the binding interface. It was found that there exist hot spots where certain residues exert major contributions to the binding energy of Hb-Hp complex [49], [50]. Particularly, Thorn and Bogan [51], [52] observed from their exhaustive collection of hot spots from alanine scanning mutagenesis data that hot spots are typically surrounded by residues with less energetic contribution to binding energy. The topology of such observation had the hotspot residue situated on the inside while its surrounding residues form the so-called O-ring. Such analysis revealed that hot spots are typically comprised of Trp, Tyr and Arg. These residues were found to be surrounded by aromatic rings whose plausible role is in the occlusion of bulk solvent [52].

In order to identify hot spots, the top ten complexes of both Hb-Hp and Hp_β_-Hp were subjected to computational alanine scanning mutagenesis using the FoldX plugin in Yasara in order to identify putative hot spot residues using 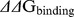 ≥1.5 kcal/mol as cut-off criteria. The results strongly suggest that βTrp37 (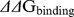  = 3.97, 2.74, 2.54, 2.25, 2.02 and 1.61 kcal/mol) and βTyr145 (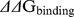  = 1.61 and 2.5 kcal/mol) are hot spots of Hb while βPhe131 (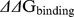  = 2.92, 2.7, 2.58, 2.54, 1.91, 1.89, 1.86 and 1.71 kcal/mol) is a hot spot of Hp as judged from a frequency of at least 2 and at most 8 models (from the top ten complex for both Hb-Hp and Hp_β_-Hp) having 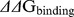 ≥1.5 for both Hb-Hp and Hp_β_-Hp complexes.

**Figure 7:**
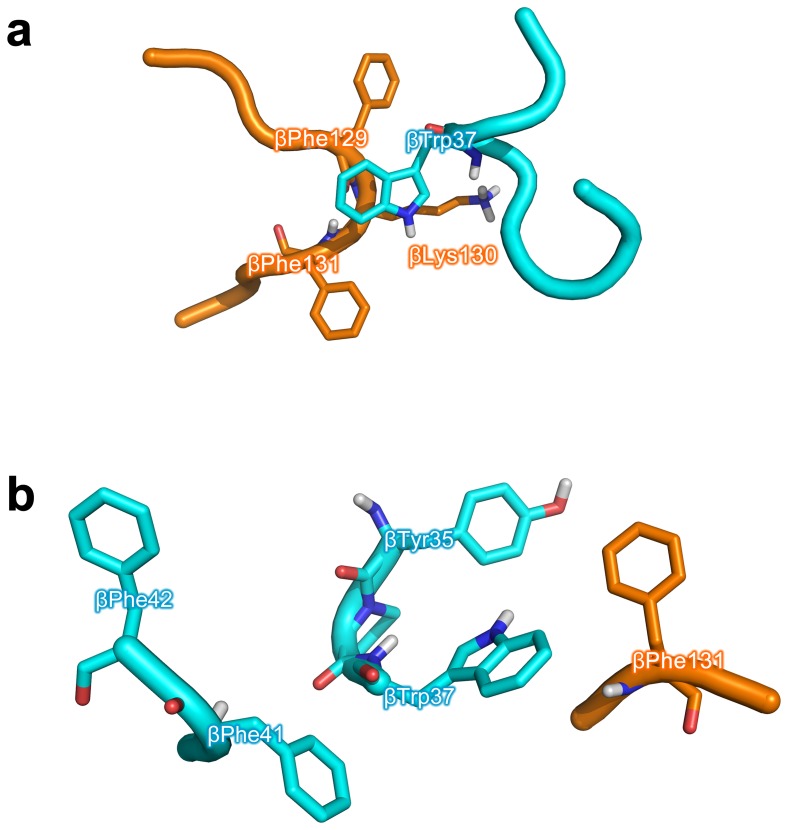
Molecular modeling analysis at the binding interface of the top ranked complex of Hb-Hp with particular focus on βTrp37 of Hb (a) and βPhe131 of Hp (b). The former panel shows βTrp37 of Hb participating in π-π ring stacking interaction with two neighboring phenylalanines of Hp, βPhe129 and βPhe131, as well as engaging in π-cation interaction with a nearby lysine of Hp, βLys130. The latter panel shows βPhe131 of Hp taking part in intermolecular π-π stacking interactions with aromatic residues of Hb, βTyr35 and βTrp37. In addition to the inner sphere of aromatic residues of Hb (comprising of βTyr35 and βTrp37), a second outer sphere of aromatic residues of Hb (comprising of βPhe41 and βPhe42) is also present.

On the basis of molecular modeling analysis on the top ranked complex of Hb-Hp, it is observed that βTrp37 of Hb participates in π-π ring stacking interaction with two neighboring phenylalanines of Hp, βPhe129 and βPhe131, as well as engaging in a possible π-cation interaction with a nearby lysine of Hp, βLys130 (Figure 7a). It is also interesting to note that both βTrp37 and βPhe131 are hot spots of each respective protein and both are interacting with one another suggesting the importance of this interacting region.

It is observed from Figure 7b that βPhe131 of Hp takes part in intermolecular π-π stacking interactions with aromatic residues of Hb, βTyr35 and βTrp37, where the distance is 4 and 3.8 Å apart. Aside from this inner sphere of aromatic residues there also exists a second outer sphere of aromatic residues comprising of two phenylalanines, βPhe41 and βPhe42, where the former is approximately 5.9 Å from βTrp37 and the latter is 9 Å from the former. It is also interesting to note that when taking protein dynamics into consideration that the peripheral phenylalanine may possibly come into closer contact with βTyr35 to form a more compact cluster of aromatic π-π stacking interactions.

The importance of π-cation interaction on protein stability [53], [54] and function [55], [56] is widely known. Burley and Petsko [57] analyzed the geometrical distances of amino groups (Lys, Arg, Asn, Gln and His) of side chains from nearby aromatic (Trp, Tyr and Phe) residues from the crystal structures of 33 proteins and their findings illuminated that favorable interaction occurs when the amino groups and aromatic residues are in the range of 3.4 and 6 Å apart. In our investigation, the distance between the side chains of βTrp37 and βLys130 was measured in PyMol to be approximately 6 Å apart, thus corroborating a possible π-cation interaction. If protein dynamics is also taken into consideration such distances may change to allow both interacting partners to be in closer proximity.

### Redox inactivation of Hb by Hp

The redox activity of Hb and its implications on hematological disorders are well known. Briefly, the reaction between Hb and hydrogen peroxide is known to form two intermediate products: (i) the ferryl heme iron, Fe(IV) = O and (ii) the Hb free radical, ^•^Hb(Fe(IV) = O). Such reactive species are involved in oxidative stress-related human diseases. The residues governing such deleterious effects are often tyrosine residues, which have been referred to as redox cofactors in human hemoglobin [58].

**Figure 8:**
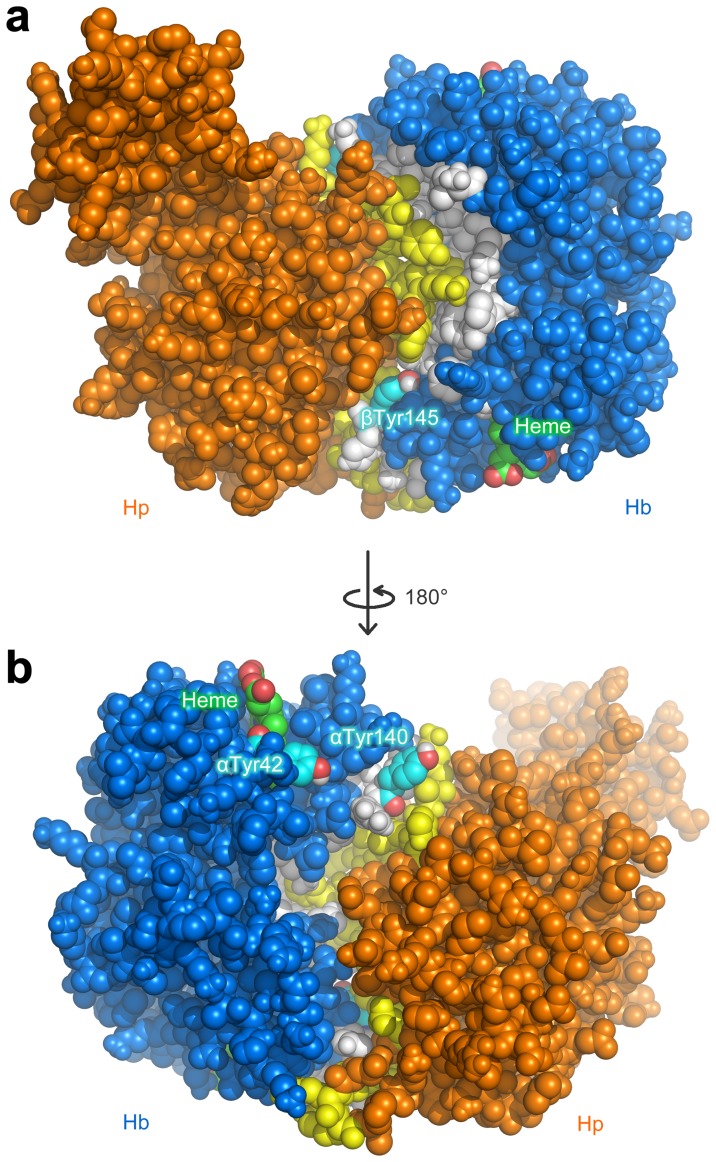
Molecular models of Hb-Hp complex as obtained from protein-protein docking calculations revealed that the penultimate tyrosine residues, βTyr145 (a) and αTyr140 (b), are situated right at the binding interface and are located on opposite side of the protein. αTyr42 of Hb (b) as well as the heme prosthetic group of both α and β-chains are located at a distance from the binding interface.

To provide a structural account on the mechanistic details of these tyrosine residues at the binding interface, a detailed molecular modeling analysis was performed. The molecular models of Hb-Hp complex as obtained from protein-protein docking calculations revealed that the penultimate tyrosine residues, βTyr145 (Figure 8a) and αTyr140 (Figure 8b), are situated right at the binding interface. Such involvement of the penultimate tyrosine implicates the possibility of electron transfer from these tyrosine residues of Hb to accepting residues of Hp. Furthermore, it was observed that αTyr42 of Hb (Figure 8b) as well as the heme prosthetic group of both the α and β-chains are located at a short distance from the Hb-Hp binding interface.

### Implication of Hp α-chain in Hb-Hp interaction

In absence of the Hp α-chain, it was observed that there was some structural perturbation that led to slightly altered protein-protein interaction. For example, the π-π interactions of βTrp37 with the two phenylalanines (βPhe129 and βPhe131) of Hp were lost while forming a new π-π interaction with βHis134. It is also interesting to note the presence of a nearby lysine (βLys136) that may potentially form π-cation interaction with the indole ring of βTrp37 as well as the presence of a distant tyrosine (βTyr137) of Hp. Nevertheless, the new interaction appears to be weaker than the former complex where the two aromatic rings of phenylalanines provided stronger stabilization. However, the removal of the α-chain brought forward a cluster of aromatic residues of Hp β-chain (βPhe12, βTyr119, βHis201 and βTrp208) onto the binding interface. Looking at αTyr42, its distance from αAsp94 increased slightly but accessible to engage in redox transfer. Thus, the loss of the Hp α-chain led to perturbation of π-π interactions of βTrp37 with βPhe129 and βPhe131. As βPhe131 has been shown to be a hot spot residue, such perturbation is thus detrimental towards the Hb-Hp complex formation. This suggests the role of the Hp α-chain in maintaining the internal structural integrity of the Hp structure where its removal led to structural rearrangement of the structure, which affected the binding with Hb.

### Comparison of modeled and crystal structure of Hb-Hp interaction

At the time of manuscript submission, the crystal structure of Hb-Hp complex was not yet available even though initial efforts attempted by Przybylska et al. [17] failed to obtain crystals that could diffract well enough for data collection. In absence of the crystal structure our study therefore represented a lucrative approach for shedding light on such important interaction. Nevertheless, the recently released crystal structure of porcine Hb-Hp complex (accession number 4F4O) by Andersen et al. [59] could thus be used as an external validation of the computational methodology described in our manuscript reminiscing that of the Critical Assessment of Predicted Interactions (CAPRI) [60], a community-wide assessment of computational methods for protein-protein docking in predicting protein-protein interactions for which its X-ray crystallographic structure has not yet been solved.

**Figure 9:**
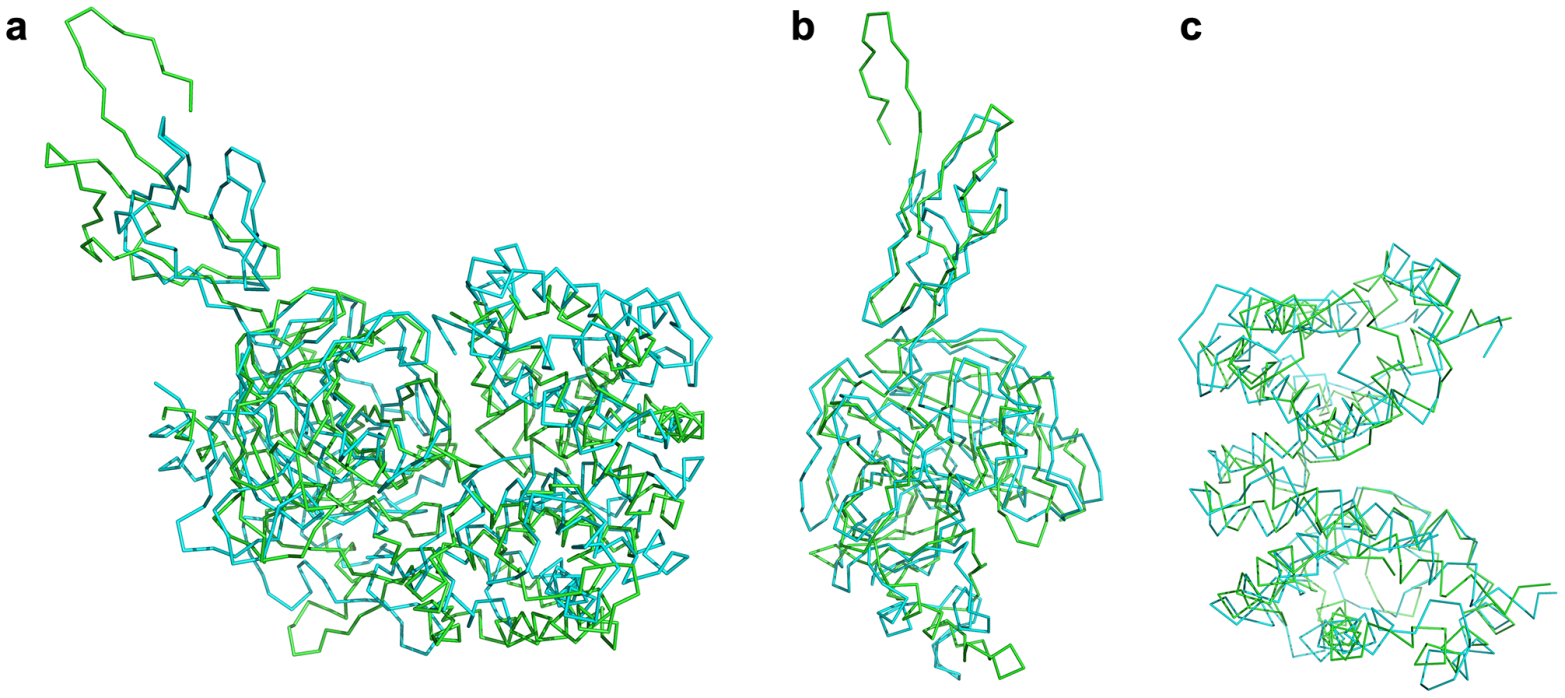
Structure superimposition of modeled (cyan) and crystal (green) structure of Hb-Hp complex (a) as well as Hp (b) and Hb (c) structures.

A comparative analysis of the modeled and crystal structure of Hb-Hp complex was performed by sequentially superimposing the crystal structure (chains A, B and C) with each of the top 10 modeled structures from HADDOCK protein-protein docking simulations. Results from superimposition of the structures indicated that models 1 through 10 had RMSD values of 10.6, 12.2, 11, 12.3, 12.5, 12.8, 12.8, 11.1, 10.9 and 12.2 Å, respectively, which had been aligned over 444, 437, 431, 433, 432, 426, 431, 443, 443 and 445 residues, respectively. It can be seen that the selected best Hb-Hp docking model or model 1 also possessed the lowest structural variation with respect to the crystal structure. Furthermore, the structure superimposition of model 1 with that of the crystal structure (Figure 9a) further corroborates the robustness of the methodology employed herein in predicting Hb-Hp interaction. It is worthy to note that the 10 top modeled structures were from the same cluster of 87 Hb-Hp docking structures with positional RMSD of 2.35 Å, which also suggests that the remaining 9 models also display similar overall binding topology as that observed from model 1.

It is observed that the modeled structure overlays nicely with the crystal structure where the Hp α-chain is positioned away from the Hb-Hp interface. Although the overall structure of both complexes provided good superimposition, the Hp α-chain did not overlay so well, which can be attributed to the truncated α-chain (13 residues missing) of the modeled structure in comparison to the crystal structure. This is in concomitant with the lack of a disulfide bridge formation between Cys15 of the Hp α-chain with that of its partner α-chain, which is not present in the model owing to the simplification made to the modeling performed herein. This may partly account for the observed greater mobility in the Hp α-chain that was detrimental towards the structure superimposition of the modeled and crystal structures. Results from structure superimposition of the Hp structure alone from the modeled and crystal structures corroborate this point, as the structural variation of Hp modeled and crystal structure (Figure 9b) was relatively high with a calculated RMSD value of 9.29 Å while the structural variation of Hb modeled and crystal structure (Figure 9c) was significantly lower with an RMSD of 3.74 Å. Nevertheless, the main features of Hb-Hp interaction as elucidated from X-ray crystallography where the (1) Hb dimer contact area in Hb tetramers overlaps with those of the Hp-binding interface of Hb as well as (2) masking of redox residues of Hb upon Hp binding were provided by the modeled structures. In regards to the latter, the two penultimate tyrosines (i.e. βTyr145 and αTyr140), which was previously mentioned to be situated at the Hb-Hp binding interface of the modeled structure were indeed present in the crystal structure where Hp were found to mask those redox residues. Likewise, αTyr42, which was previously mentioned to be at a short distance from the binding interface were also present in the crystal structure at a similar distance away from the binding interface.

In comparison to the crystal structure of the porcine Hb-Hp complex, residues βPhe129, βLys130 and βPhe131 of Hp from the human Hb-Hp complex (Figure 7a) was found to be different from the porcine complex where the former two residues were Leu and Asn instead of Phe and Lys of the human complex. In regards to βTrp37 as found in the human complex, Trp was also found at a comparable position of Hp from the porcine complex. All amino acid residues present at the aromatic cluster as shown in Figure 7b were found to be the same in both human and porcine complex. It should be noted that in the crystal structure of the porcine complex, the Phe residue from Hp of the porcine complex (i.e. corresponding to that of βPhe131 from Hp of the human complex) swings away from the aromatic clusters from Hb of the porcine complex when compared to Hb from the human complex. However, protein dynamics may allow the Phe residue from Hp of the porcine complex to swing out from the protein cavity and engage in interaction with the aromatic cluster.

## Conclusions

Hb unleashes its reactivity and toxicity upon hemolysis. Hp counteracts the deleterious effects of Hb by binding and relaying Hb for clearance via CD163-mediated endocytosis by macrophages. The interaction of Hb and Hp represents one of the strongest interactions known in nature and the structural details of such binding have yet to be fully elucidated. With the great advancements in molecular modeling it is possible to shed light on this important interaction through the use of several computational approaches. The initial structures of Hb and Hp was subjected to a steepest descent energy minimization their energetically favorable conformation was obtained by means of a preliminary steepest descent energy minimization and MD simulation. Finally, an ensemble of structures from the final 10 ns of MD simulation was cross-docked to yield a subsequent set of possible Hb-Hp complex. Post-analysis of these top ten complex provided pertinent information on the binding interface of Hb-Hp and Hb-Hp_β_, particularly that their interaction surface is not flat and protrudes well into each binding partner. Furthermore, it was also observed that the secondary structure at the interface takes the form of coils and α-helices. The results obtained herein are consistent with previously reported data on Hb and Hp binding. The robustness of the computational methodology described herein is further corroborated by the good superimposition of the modeled and crystal structures of Hb-Hp complexes. Such information provided structural insights on the binding modalities of Hb-Hp, which may be useful in the design of Hp mimics as Hb scavengers.
